# Glycaemic, appetite and circadian benefits of a dairy-enriched diet with high-protein breakfast and early daytime-restricted carbohydrate intake in type 2 diabetes: a randomised crossover trial

**DOI:** 10.1007/s00125-025-06658-2

**Published:** 2026-01-23

**Authors:** Shani Tsameret, Oren Froy, Yael Matz, Zohar Landau, Orit Twito, Julio Wainstein, Natalie Avital-Cohen, Nava Chapnik, Daniela Jakubowicz

**Affiliations:** 1 Institute of Biochemistry, Food Science and Nutrition, The Robert H. Smith Faculty of Agriculture, Food and Environment, The Hebrew University of Jerusalem, Rehovot, Israel; 2 Endocrinology and Diabetes Unit, Wolfson Medical Center, Gray School of Medical Sciences, Tel Aviv University, Holon, Israel

**Keywords:** CGM, Circadian clock, Dairy, Glycaemic management, GMI, Meals

## Abstract

**Aims/hypothesis:**

The circadian timing of food intake and the composition of dietary protein sources may jointly influence metabolic regulation. Our aim was to examine the effects of a dairy-enriched vs non-dairy isoenergetic diet with structured meal timing on circadian clock gene expression, glycaemic management and appetite regulation in individuals with type 2 diabetes.

**Methods:**

In a randomised, crossover trial, 25 participants with type 2 diabetes and HbA_1c_ ≥48 mmol/mol (6.5%), treated either with stable doses (≥3 months) of oral glucose-lowering agents or managed by diet, followed two 4 week dietary phases, one including dairy-based protein sources (YesMilk) and one excluding them (NoMilk), with a 3–4 week washout. Participants were randomly assigned to one of two intervention sequences using simple randomisation (coin flip), either starting with the YesMilk diet followed by the NoMilk diet, or vice versa. Due to the open-label design, allocation was not concealed from investigators or participants. The study was powered for the primary outcome of circadian clock gene expression in peripheral blood mononuclear cells. Secondary outcomes included glycaemic indices derived from continuous glucose monitoring (CGM) and appetite scores. The study was conducted via the Diabetes Unit at Wolfson Medical Center, Israel.

**Results:**

Twenty-nine individuals were screened; 25 met eligibility criteria and were randomised to YesMilk or NoMilk dietary interventions in a crossover design. Thirteen participants began with the YesMilk dairy diet, all of whom completed both phases. Of the 12 who began with the NoMilk diet, six completed the study. Nineteen participants completed both intervention phases. Compared with the NoMilk phase, the YesMilk diet upregulated *BMAL1* (+1.8-fold, *p*=0.0003), *REV-ERBα* (also known as *NRD1D1*) (+2.2-fold, *p*<0.001) and *CRY1* (+1.4-fold, *p*=0.03), with higher *PER1* expression (*p*=0.01 between diets at 4 weeks). Glycaemic variables improved under the YesMilk diet, with fasting glucose reduced by ~1.7 mmol/l, glucose management indicator reduced by 0.7%, and time in range increased by 9% compared with baseline (all *p*<0.05). Hunger and sweet craving scores decreased by 15–20% (*p*<0.05).

**Conclusions/interpretation:**

A dairy-enriched diet aligned with structured meal timing enhanced circadian clock gene expression and improved glycaemic and appetite-related variables in individuals with type 2 diabetes. These findings support a mechanistic link between dietary protein source, circadian regulation and metabolic health, warranting confirmation in larger, long-term studies.

**Trial registration:**

ClincalTrials.gov NCT03772067

**Funding:**

The Israeli Ministry of Health provided funding.

**Graphical Abstract:**

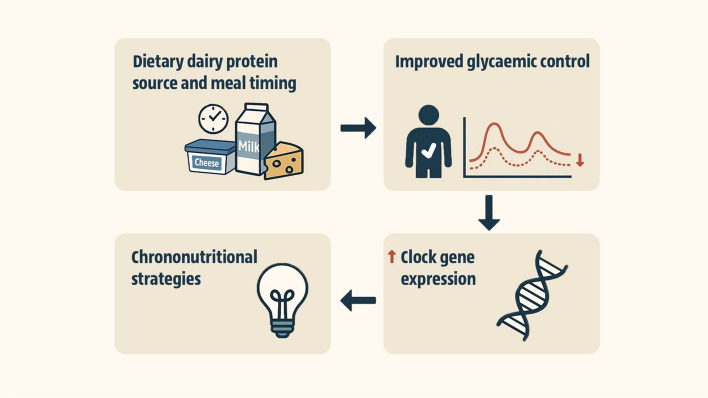

**Supplementary Information:**

The online version contains peer-reviewed but unedited supplementary material available at 10.1007/s00125-025-06658-2.


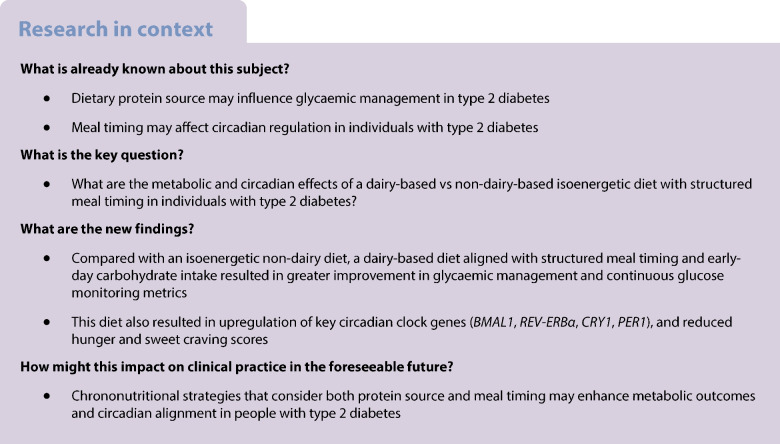


## Introduction

Type 2 diabetes is characterised by progressive beta cell dysfunction and insulin resistance, resulting in elevated postprandial glucose levels and increased glycaemic variability, major contributors to higher HbA_1c_ and cardiovascular risk [1]. Minimising glycaemic excursions is therefore a key therapeutic goal. Recent studies highlight the role of the circadian system in regulating glucose uptake, insulin sensitivity, beta cell function, hepatic glucose production and hormone secretion [2, 3]. Synchrony between the central clock (entrained by light) and peripheral clocks (primarily entrained by food timing) is essential for optimal glucose homeostasis [4] and disruption of this alignment may contribute to type 2 diabetes development [5].

At the molecular level, the circadian clock consists of feedback loops driven by the circadian locomotor output cycles kaput (CLOCK)–brain and muscle ARNT-like protein 1 (BMAL1) heterodimer, which activates the expression of periodic circadian regulators (PERs), cryptochromes (CRYs), nuclear receptor subfamily 1 group D (REV-ERBs) and retinoic acid receptor-related orphan receptors (RORs). These proteins modulate tissue-specific processes, such as insulin secretion, fat metabolism and muscle glucose uptake [2, 6, 7]. In type 2 diabetes, disrupted clock gene expression has been associated with impaired insulin secretion [8]. Eating patterns misaligned with circadian rhythms can further worsen this dysregulation, leading to poor glycaemic management [9]. In contrast, concentrating energy and carbohydrate intake earlier in the day can improve glycaemic management, insulin sensitivity and weight loss and restore circadian clock gene expression [10–13].

Protein source may influence metabolic outcomes. Dairy proteins are linked to improved glycaemic management, appetite suppression, lower body weight, increased insulin sensitivity and reduced risk of obesity and type 2 diabetes [14–17]. When consumed with carbohydrates at breakfast, dairy proteins elicit stronger postprandial insulin and glucagon-like peptide-1 (GLP-1) responses and greater ghrelin suppression than other proteins, enhancing glucose management and appetite regulation [14, 18–20]. Amino acids and bioactive peptides in dairy products may also entrain peripheral circadian clocks and modulate gene expression [21, 22]. Dairy-derived peptides inhibit dipeptidyl peptidase-4, preserving incretin activity and supporting insulin secretion and appetite regulation [23]. Additionally, dairy intake stimulates IGF-1, which upregulates PER1/2 and improves beta cell function [22, 24]. Despite these findings, the impact of protein source on glycaemic indices and circadian gene expression remains underexplored in clinical settings.

While general dietary guidelines recommend three daily dairy servings [25], type 2 diabetes-specific guidelines provide no quantitative recommendations or distinctions by protein type. The ADA advises including low-fat dairy within a healthy diet but emphasises individualised nutrition without specifying protein type or amount. These gaps, alongside emerging evidence on dairy benefits, underscore the need for research on dairy protein effects on glycaemic management and circadian gene expression in type 2 diabetes. This study compared a high-dairy-protein diet with a non-dairy isoenergetic alternative featuring a high-protein breakfast and daytime-restricted carbohydrates, assessing impacts on clock gene expression, glycaemic outcomes and appetite regulation.

## Methods

### Study population

The study included 29 individuals (15 women and 14 men) aged 45–77 years, with a diagnosis of type 2 diabetes and HbA_1c_ ≥48 mmol/mol** (**6.5%). All were treated with stable doses (≥3 months) of oral glucose-lowering agents or were managed by diet. Additional inclusion criteria included stable weight (<5% change in the previous 3 months), normal liver, kidney and thyroid function, and eGFR>60 ml/min per 1.73 m^2^. Concomitant medications (e.g. antihypertensive or lipid-lowering agents) were permitted if doses were stable for at least 3 months. Participants maintained a regular sleep–wake schedule (waking at 06:00–08:00 hours and sleeping at 22:00–24:00 hours). Exclusion criteria included shift work within 6 months of enrolment or recent travel across more than two time zones, type 1 diabetes, insulin therapy, active malignancy, use of psychotropic/steroid medications, substance abuse, pregnancy/lactation and known hypersensitivity to milk components. Participants were instructed to maintain their usual physical activity throughout the study. Recruitment was conducted via the Diabetes Unit at Wolfson Medical Center, regional diabetes conferences and support group meetings. All participants were Israeli, but detailed data on ethnicity, religious background and socioeconomic factors were not collected. Gender was self-reported and verified *via* the medical record. The study protocol was approved by the Helsinki Ethics Committee of Wolfson Medical Center (Holon, Israel) and written informed consent was obtained from all participants prior to enrolment. Recruitment began in late 2019, was paused due to the COVID-19 pandemic, and resumed after protocol amendments and re-approval. Recruitment was completed by July 2024 and the last visit occurred in October 2024. The study was registered at ClinicalTrials.gov (registration no. NCT03772067).

### Study design

**Fig. 1:**
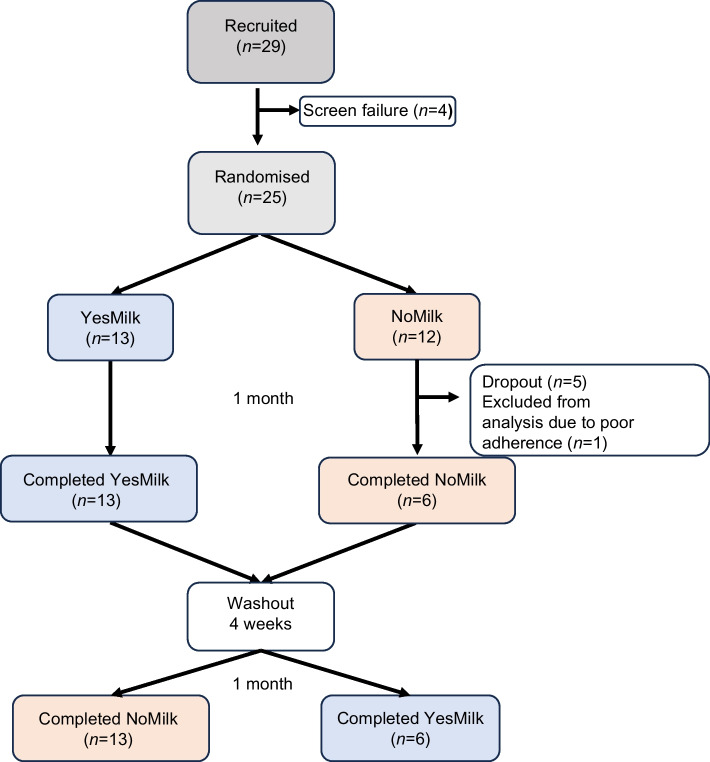
Study flowchart

This was an open-label, randomised, two-period crossover clinical trial conducted over approximately 12 weeks per participant. The study included a baseline screening phase, two 4 week dietary intervention periods and a 3–4 week washout period between interventions. Participants were randomly assigned to one of two intervention sequences using simple randomisation (coin flip), either starting with the YesMilk diet (dairy-based protein sources) followed by the NoMilk diet (excluding dairy-based protein sources), or vice versa (Fig. 1). Given the open-label design, allocation was not concealed from investigators or participants. The randomisation sequence was generated by the study physician, who also enrolled participants and assigned them to the intervention sequence. Individual energy requirements were calculated prior to randomisation. Participants received detailed dietary instructions and were followed up every 2 weeks by a registered dietitian and a physician at the clinic, and at least twice a week by telephone calls. Eight in-person follow-up visits were held at the Wolfson Medical Center. At each visit, anthropometric measurements were recorded and dietary adherence was assessed. Continuous glucose monitoring (CGM) was performed throughout the study. Venous blood samples were collected at screening, baseline (visits 3 and 6), after 2 weeks of each dietary phase (visits 4 and 7) and after 4 weeks (visit 5 and 8) of each phase. This corresponded to three blood collections per intervention phase (a total of seven per participant). Subjective appetite scores were assessed weekly using a validated visual analogue scale (VAS) questionnaire. A minimum 3 week washout period was implemented between dietary phases to minimise potential carry-over effects.

### Diet intervention

Both dietary interventions were isoenergetic with matched macronutrient content, meal timing and carbohydrate distribution, following a previously described protocol [26]. Energy intake was distributed as follows: a high-energy, carbohydrate-rich breakfast (45% of total intake); a large lunch (40% of total intake); and a low-energy, carbohydrate-free dinner (15% of total intake) (electronic supplementary material [ESM] Table 1). Carbohydrates were limited to breakfast and lunch; dinner carbohydrates were minimal and derived mainly from non-starchy vegetables (mostly attributed to dietary fibre). Calcium and fibre intake was comparable across both diets. The sole difference between diets was the protein source. Participants prepared their own meals at home according to individualised meal plans specifying exact food quantities, portion size and timing. All ingredients were purchased and prepared by the participants under twice-a-week supervision and dietary counselling by a registered dietitian. The YesMilk diet emphasised dairy-based protein sources, including at least one serving of fluid milk at breakfast, and additional dairy products throughout the day. The NoMilk diet included only non-dairy protein sources (eggs, poultry, fish, meat, legumes). A soy-based milk alternative was included in the meal plan at breakfast to match the fluid dairy component of the YesMilk diet. Prior to randomisation, a dietary anamnesis was performed by a registered dietitian and individual energy needs were estimated. Participants received dietary instructions aligned with an energy intake of approximately 7500–9200 kJ/day (1800–2200 kcal/day) (ESM Table 1).

### Compliance and adherence to the diet

During the first 2 weeks of each intervention phase, participants were closely monitored to ensure weight stability. Energy intake was adjusted as needed to maintain energy balance. Participants received tailored written guidelines detailing daily food quantities, individualised meal plans and a substitution list with energetic and nutritionally equivalent alternatives. They were instructed to record their daily food intake and meal timing, which were reviewed every 2 weeks at clinic visits. At least two weekly phone calls were conducted to assess real-time dietary intake. Dietary intake was assessed through food diaries, 24 h recalls, telephone interviews and in-person anamnesis. All dietary records were analysed using the Tsameret dietary software (Israeli Ministry of Health), based on national food composition data. Food-group servings were categorised according to national dietary guidelines. A second registered dietitian independently reviewed all records. Non-compliance was defined as follows: (1) >10% deviation from prescribed energy intake per meal/day; (2) failure to follow meal timing schedule or macronutrient distribution; or (3) consumption of a protein source inconsistent with the assigned diet. Participants non-compliant for >3 days/week were excluded from the study.

### CGM

CGM was performed using FreeStyle Libre-2 sensors (Abbott, Alameda, CA, USA) applied at baseline, washout and every 2 weeks during each dietary phase (6 periods). A minimum of four complete 24 h recording days per phase was required for inclusion in the final data analysis, consistent with previous CGM studies [26, 27]. CGM-derived metrics included fasting glucose, 24 h mean glucose, glucose management indicator (GMI), time in range (TIR), and daily glucose fluctuations. Standard metrics, such as TIR and 24 h mean glucose, were calculated using the LibreView software (Abbott). In addition, a semi-automated algorithm developed specifically for this study was used to identify fasting glucose and postprandial glucose excursions (defined as ≥2.2 mmol/l increase from baseline) within the following predefined meal windows: 08:00–12:00 hours (breakfast); 12:00–16:00 hours (lunch); and 17:00–21:00 hours (dinner). The earliest daily increase per window was selected, according to previous CGM studies [28]. Outputs from our algorithm were cross-validated against metrics generated by Libre-derived data (ESM Fig. 1).

### Analysis of gene expression

Peripheral blood was collected in Tempus RNA tubes (Applied Biosystems, Foster City, CA, USA), and total RNA was extracted according to the manufacturer’s instructions. One microgram of RNA was reverse-transcribed using the High-Capacity cDNA Reverse Transcription Kit (Applied Biosystems). Quantitative PCR (qPCR) was performed using SYBR Green Master Mix (Thermo Fisher Scientific) on a QuantStudio 6 system. Relative expression was calculated using the ${2}^{-\Delta \Delta {\mathrm{C}}_{\mathrm{t}}}$ method and normalised to the reference gene actin. Candidate genes were selected a priori based on their central role in circadian regulation and metabolic control: *BMAL1*, *REV-ERBα* (also known as *NR1D1*), *CRY1* and *PER1*. These genes represent key components of the core transcriptional–translational feedback loop of the circadian clock. Primer sequences spanning exon–exon boundaries are listed in ESM Table 2 as was previously reported [26]. Additional clock-related genes (*CLOCK* and *RORα* [also known as *RORA*]) were also examined but did not show statistically significant differences between diets. The primary outcome of the study was the change in circadian clock gene expression (*BMAL1*, *REV-ERBα*, *CRY1*, *PER1*) between the YesMilk and NoMilk dietary phases.

### Appetite and craving questionnaires

Appetite was assessed using validated 100 mm VAS, anchored by ‘not at all’ (0 mm) and ‘extremely’ (100 mm). Participants completed the VAS hourly from waking to bedtime, once weekly during baseline and once weekly during each dietary phase. Thus, each participant completed the full-day VAS assessment at baseline and four times for each period (YesMilk and NoMilk). This instrument has been previously validated for free-living circadian appetite monitoring [29].

### Sample size and power analysis

The primary outcome was the change in circadian clock gene mRNA expression in response to the dietary interventions; this guided the sample size calculation. Secondary outcomes included glycaemic indices derived from CGM and subjective appetite scores. A sample size of *n*=19 participants completing both interventions was estimated to provide ≥80% power to detect a moderate-to-large within-participant difference of approximately 50%±30% in clock gene expression (α=0.05), based on prior data [10]. Post hoc analysis demonstrated >99% power to detect >50% differences in *REV-ERBα* and *PER1* expression. The study was powered based on expected within-participant differences in circadian clock gene expression (primary outcome), as described above. Glycaemic and appetite outcomes were analysed as secondary endpoints without separate power estimation. To account for an anticipated dropout rate of ~20%, we aimed to recruit at least 24 participants. Twenty-nine individuals were screened and 25 were randomised, yielding an attrition rate consistent with similar studies [11, 26]. No changes were made to the primary or secondary outcomes after trial commencement.

### Statistical analysis

Statistical analyses were performed using JMP Pro (18.0.2, SAS Institute, Cary, NC, USA). All analyses followed a per-protocol approach and included only participants who completed both dietary intervention phases (*n*=19). Given the crossover design, each participant served as his/her own control, ensuring complete paired data across conditions. Data distribution for each variable was assessed using histograms, boxplots and normal Q–Q plots. Homogeneity of variances was evaluated using Levene’s and Bartlett’s tests to guide selection of appropriate tests. Since this was a crossover design in which each participant served as their own control, all within- and between-diet comparisons were analysed using paired Student’s *t* tests (or paired Welch’s *t* tests when variances were unequal). Welch’s *t* test was used to compare means between groups, accounting for unequal variances; otherwise, Student’s *t* test (pooled variance) was applied. Tukey’s HSD test was applied for multiple time point comparisons within and between treatment arms. For time series, a one-way ANOVA was performed to analyse daily or 24 h patterns. A post hoc *t* test analysis was used for comparison between the YesMilk and NoMilk groups at each time point. A *p* value of <0.05 was considered statistically significant. Data are presented as mean ± SEM for outcome variables (unless stated otherwise) and mean ± SD for baseline characteristics. Linear mixed-model (LMM) analyses were performed, including treatment, time, period, and their interaction as fixed effects, and participant as a random intercept. Clock gene expression values were log-transformed prior to analysis. Full model outputs are presented in ESM Table 3. A significant period effect was observed for TIR and time above range (TAR) in the exploratory LMM, likely reflecting sequence imbalance and variability in CGM-derived data rather than biological carry-over. However, the absence of significant treatment × time interactions and the consistency with paired analyses substantiate the validity of the main results.

## Results

### Participants

Twenty-nine individuals with type 2 diabetes were screened, 25 met eligibility criteria and were randomised to one of two dietary interventions in a crossover design. Thirteen participants began with the YesMilk dairy diet, all of whom completed both phases. Of the 12 who began with the NoMilk non-dairy diet, six completed the study, five withdrew due to difficulty avoiding dairy, and one was excluded for low adherence. In total, 19 participants (8 women, 11 men) completed both interventions (Fig. 1). Baseline characteristics (Table 1) were comparable between interventions and no adverse events were reported.

**Table 1:** Participant characteristics at baseline

Characteristic	*N*=19
Gender, *n* female/*n* male	8/11
Age, years	58.1±9.9 (45–77)
Type 2 diabetes duration, years	10±6.06 (1–20)
Weight, kg	90.8±4.7 (61.6–132.7)
BMI, kg/m^2^	31.02±5.4 (22.85–41.8)
Pulse rate, beats/min	82.32±16 (64–135)
Systolic BP, mmHg	126.6±27 (47–173)
Diastolic BP, mmHg	85.2±24.4 (60–177)
HbA_1c_, mmol/mol	57.7±3 (48–104)
HbA_1c_, %	7.4±0.28 (6.5–11.7)
Fasting glucose, mmol/l	8±1.0 (7.1–10.7)
Fasting insulin, mmol/l	1.1±0.4 (0.6–2.1)
Alanine aminotransferase (ALT), U/l	31.6±17 (11–79)
Aspartate aminotransferase (AST), U/l	23.5±8 (11–39)
Creatinine, µmol/l	65.6±19.1 (38.9–100.7)
C-reactive protein (CRP), mg/l	4.9±4.5 (1–17.4)
Haemoglobin, g/l	147±136 (128–172)

Data are presented as mean ± SD (range)

### Dietary intake

Dietary intake is summarised in ESM Table 4. Baseline food intake showed no differences in energy or macronutrient intake between the pre-YesMilk and pre-NoMilk conditions (*p*>0.05) (ESM Table 5). The YesMilk and NoMilk diets were isoenergetic and matched in macronutrient distribution and carbohydrate allocation per meal (ESM Tables 4, 5). The only notable difference was in calcium distribution, which was higher at breakfast in the NoMilk group and higher at dinner in the YesMilk group (*p*<0.05, between diets) (ESM Table 6), while total daily calcium intake remained comparable between diets (*p*=0.91) (ESM Table 4). Participants in the YesMilk group consumed approximately five servings of dairy protein per day, including ~1.5 servings of fluid dairy and ~3.5 servings of other dairy products (ESM Table 7). They also consumed ~5.5 servings of non-dairy protein, compared with ~10.5 servings in the NoMilk group, reflecting the substitution of dairy with other protein sources.

Meal composition analysis of the YesMilk diet intervention showed that ~73% of daily dairy protein intake occurred at breakfast, which consistently included at least one serving of liquid dairy products and was therefore rich in whey protein (ESM Table 8). Lunch contributed only ~4% of daily dairy protein intake, with protein intake at lunch being mostly non-dairy due to local cultural practices. The remaining daily dairy protein intake (~23%) was consumed at dinner, a carbohydrate-free meal composed mainly of casein-rich, low-lactose dairy products with minimal whey content.

### Expression of circadian clock genes

**Fig. 2:**
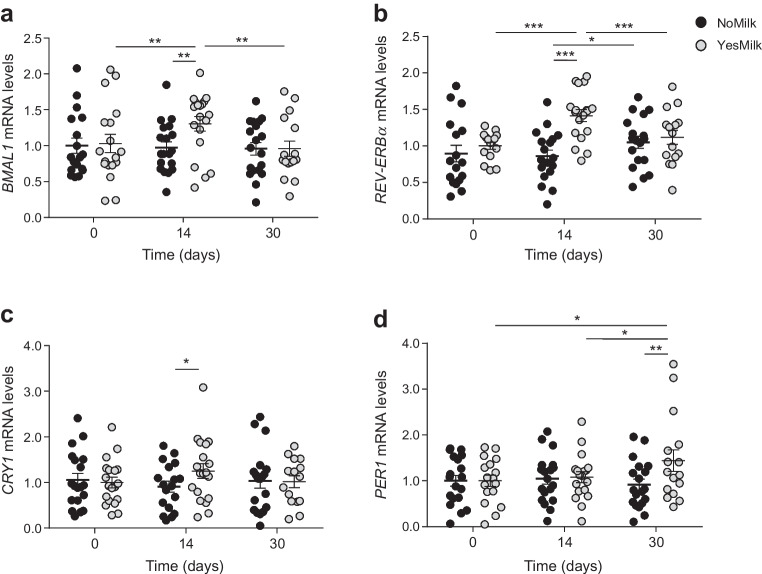
Effects of dietary protein source on circadian clock gene expression. mRNA levels of core clock genes measured in white blood cells in the fasting state, at baseline and at 2 weeks and 1 month. (**a**) *BMAL1*, (**b**) *REV-ERBα*, (**c**) *CRY1* and (**d**) *PER1* mRNA levels, comparing NoMilk (black) and YesMilk (grey) groups. Genes were normalised to the housekeeping gene β-actin and are presented as fold change relative to baseline. Each participant served as their own control. Data are presented as mean ± SEM. Statistical significance was determined using paired Student’s *t* test. *p<0.05, **p<0.01, ***p<0.001

As the prespecified primary outcome, expression of the core clock genes *BMAL1*, *REV-ERBα*, *CRY1* and *PER1* was measured in the fasting condition in white blood cells at baseline, and at 2 and 4 weeks of diet. After 2 weeks on the YesMilk diet, *BMAL1* and *REV-ERBα* expression increased vs baseline (*BMAL1 p*=0.0036, *REV-ERBα p*<0.001), with no changes in the NoMilk group, yielding statistically significant between-diet differences (*BMAL1 p*=0.0003, *REV-ERBα p*<0.001, *CRY1 p*=0.03) (Fig. 2a–c). By week 4, these genes returned to baseline levels. In contrast, *PER1* expression was upregulated at week 4 under the YesMilk diet (*p*=0.037 within-diet, *p*=0.01 between diets) (Fig. 2d). This pattern indicates early induction of *BMAL1*, *REV-ERBα* and *CRY1*, followed by delayed activation of *PER1*.

### Glycaemic regulation and body weight maintenance

**Fig. 3:**
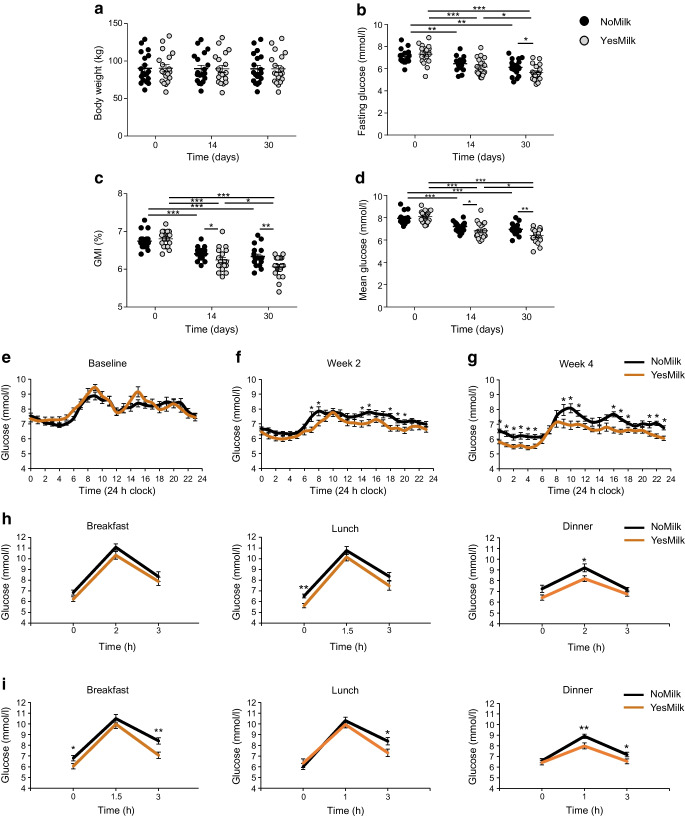
Clinical and CGM outcomes following dietary interventions. The effects of a dairy-protein-based diet (YesMilk) vs a non-dairy protein diet (NoMilk) on glycaemic regulation and body weight over 30 days. (**a**) Body weight (kg). (**b**) Fasting glucose levels (mmol/l). (**c**) GMI (%). (**d**) Mean glucose levels (mmol/l). (**e**–**g**) Diurnal glucose profiles (mmol/l) across 24 h periods at baseline (**e**), 2 weeks (**f**) and 1 month (**g**). (**h**, **i**) Pre- and postprandial glucose responses by meal and time point after 2 weeks of diet (**h**) and after 1 month of diet (**i**), measured throughout breakfast, lunch and dinner, in the NoMilk (black) and YesMilk (orange) groups. Data are presented as mean ± SEM. Statistical significance was determined using paired Student’s *t* test. *p<0.05, **p<0.01, ***p<0.001

Body weight remained unchanged in both groups throughout the intervention (Fig. 3a). Fasting glucose levels improved in both groups, more markedly in the YesMilk, with a 1.1 mmol/l reduction after 2 weeks and 1.7 mmol/l at 1 month (*p*<0.001, within-diet, vs baseline) (Fig. 3b). This represented an approximately twofold greater reduction than that observed in the NoMilk group (0.9 mmol/l, *p*=0.0002, within-diet, week 4 vs baseline), with a statistically significant between-diet difference at day 30 (*p*=0.045, between diets, week 4) (Fig. 3b).

The GMI, which estimates laboratory-measured HbA_1c_ from CGM data, decreased in both groups (Fig. 3c). YesMilk resulted in a greater reduction, from 6.8% to 6.25% after 2 weeks (*p*<0.001, within-diet, week 2 vs baseline), and to 6.06% at four weeks (*p*<0.001, within-diet, week 4 vs baseline), with statistically significant between-group differences at both time points (*p*=0.044 and *p*=0.0036, respectively, between diets, week 4) (Fig. 3c). Mean 24 h daily glucose levels declined in both groups (Fig. 3d). NoMilk mean glucose levels decreased from 7.9 mmol/l to 7.2 mmol/l after 2 weeks (*p*<0.001, within-diet, week 2 vs baseline), and to 6.9 mmol/l at 4 weeks (*p*=0.1, within-diet, week 4 vs week 2). YesMilk resulted in greater reductions, from 8.1 mmol/l to 6.8 mmol/l after 2 weeks (*p*<0.001, within-diet, week 2 vs baseline), and to 6.4 mmol/l after 4 weeks (*p*=0.05, within-diet, week 4 vs week 2). Between-group differences were statistically significant at both time points (*p*=0.0389 at week 2 and *p*=0.0043 at week 4). These outcomes were independently confirmed using a semi-automated algorithm developed specifically for this study (ESM Fig. 1).

Daily glucose fluctuations (24 h mean ± SEM) were similar at baseline, showing rhythmic daily fluctuations (*p*<0.0001) (Fig. 3e). After 2 weeks, glucose levels were lower in the YesMilk group than in the NoMilk group in the early morning (07:00–08:00 hours), early afternoon (14:00–15:00 hours) and early evening (18:00–20:00 hours) (*p*<0.05 between diets, at week 2) (Fig. 3f), with more pronounced reductions after 4 weeks in the mid-morning (09:00–11:00 hours), post-lunch (16:00–18:00 hours) and across the evening and night period (21:00–05:00 hours) (*p*<0.05 between diets, at week 4) (Fig. 3g). Both diet interventions maintained the rhythmic glucose fluctuations throughout the study (*p*<0.0001). The YesMilk diet improved glycaemic management not only after breakfast but also more prominently after lunch, dinner and during night-time. Glucose levels further decreased at 16:00–17:00 hours, 22:00–02:00 hours, and 03:00–05:00 hours by week 4 (*p*<0.05 within-diet, week 2 vs week 4 and *p*<0.0001 at week 4 vs baseline), with no notable changes in the NoMilk group (ESM Fig. 2a, b).

Pre- and postprandial glycaemic response analyses showed differences between the interventions, as pre-breakfast glucose levels were lower in the YesMilk group than in the NoMilk group, approaching statistical significance at 2 weeks (*p*=0.06, between diets) (Fig. 3h). This difference became statistically significant at 4 weeks (*p*=0.046, between diets) (Fig. 3i), congruent with the fasting glucose outcomes (Fig. 3b). Pre-lunch glucose levels were also lower at 2 weeks (*p*=0.001, between diets), with a near-significant difference before dinner (*p*=0.055, between diets) (Fig. 3h). Post-dinner glucose levels were ~1.1 mmol/l lower with the YesMilk diet than with the NoMilk diet at week 2 (*p*=0.034, between diets) (Fig. 3h). By week 4, the YesMilk group consistently showed lower glucose levels than the NoMilk group at the end of the postprandial window (3 h) after breakfast, lunch and dinner (*p*=0.003,* p*=0.033, *p*=0.053, respectively, between diets) (Fig. 3i). The most consistent and robust glycaemic improvement was observed during dinner (i.e. peak and late postprandial glucose values were statistically significantly reduced at both time points) (Fig. 3i).

### TIR and glycaemic variability

**Fig. 4:**
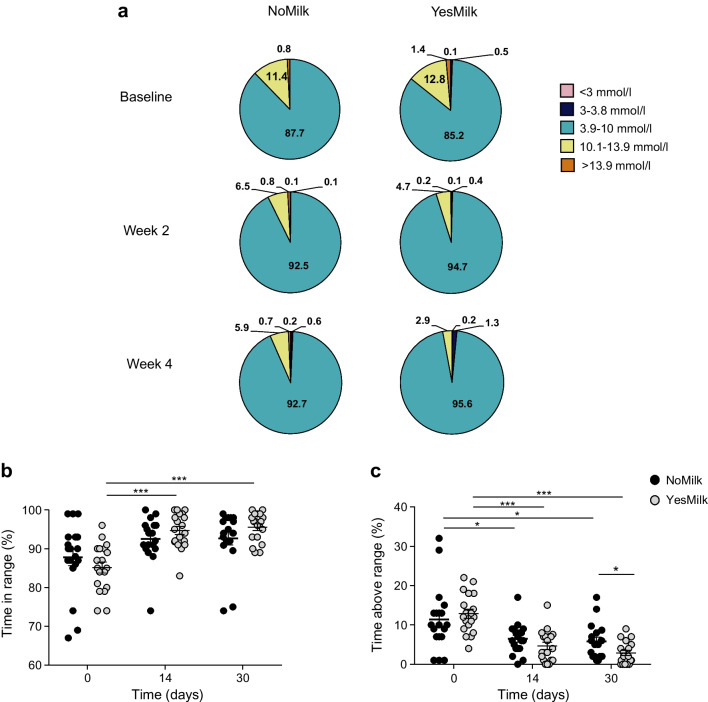
TIR and glycaemic variability. (**a**) Percentage of time spent in glucose ranges at baseline and at 2 weeks and 1 month, presented as pie charts. (**b**) TIR 3.9–10 mmol/l (%) over the study period by diet group. (**c**) TAR 10.1–13.9 mmol/l (%) over the study period by diet group. Data are presented as mean ± SEM. Statistical significance was determined using paired Student’s *t* test. *p<0.05, ***p<0.001

CGM analysis revealed a statistically significant increase in time spent in normoglycaemia (3.9–10 mmol/l) in the YesMilk group at 2 weeks (*p*=0.0001, within-diet, week 2 vs baseline), with further improvement (9%) by 4 weeks (*p*<0.001, within-diet, week 4 vs baseline) (Fig. 4a–c). The NoMilk group showed a similar pattern but the changes did not reach statistical significance (Fig. 4b). At 4 weeks, TAR (10.1–13.9 mmol/l) decreased from 12.8% to 2.9% in the YesMilk group (*p*<0.001, within-diet, week 4 vs baseline), and from 11.4% to 5.9% in the NoMilk group (*p*=0.015, within-diet, week 4 vs baseline), leading to a statistically significant between-group difference at 4 weeks (*p*=0.0227, between diets, at week 4) (Fig. 4c).

### VAS appetite scores

**Fig. 5:**
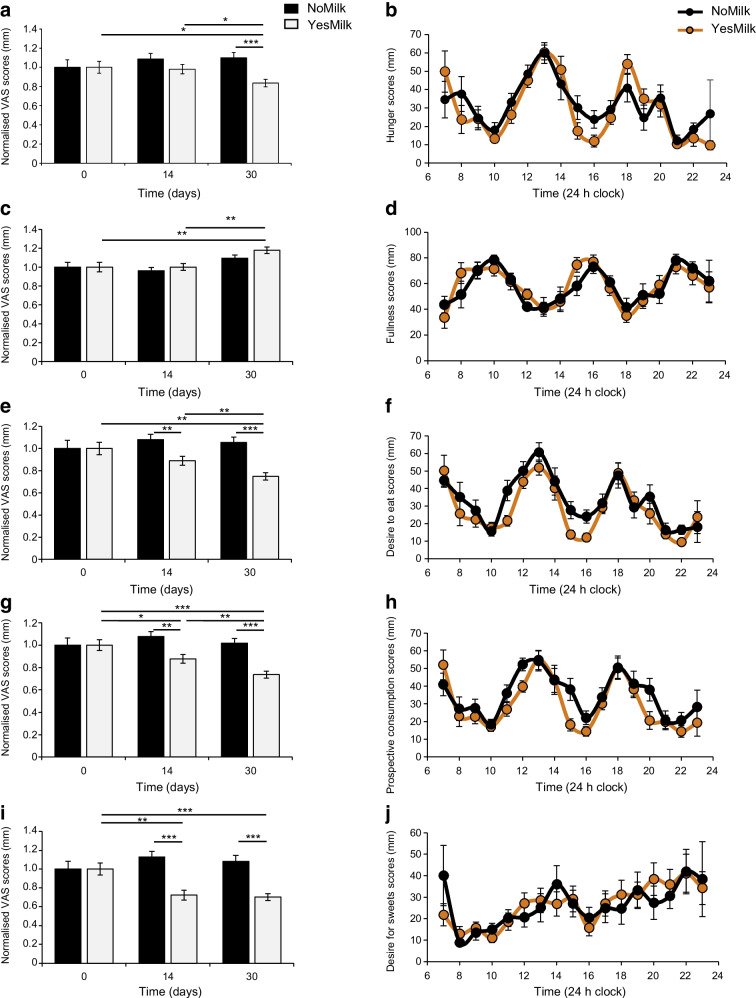
Effects of YesMilk vs NoMilk diets on subjective appetite ratings. VAS scores were assessed at baseline and at 2 weeks and 1 month, and across 24 h profiles after 1 month. (**a**, **c**, **e**, **g**, **i**) Normalised VAS scores for hunger (**a**), fullness (**c**), desire to eat (**e**), prospective consumption (**g**) and desire for sweets (**i**) are shown across time points. (**b**, **d**, **f**, **h**, **j**) Diurnal VAS profiles for the same ratings, respectively, over a 24 h period after 1 month of intervention. Black bars and circles, NoMilk group; light grey bars and orange circles, YesMilk group. Data are presented as mean ± SEM. Statistical significance was determined using paired Student’s *t* test. *p<0.05, **p<0.01, ***p<0.001

Subjective appetite ratings, normalised to baseline, demonstrated favourable responses among participants following the YesMilk diet after 1 month (Fig. 5 and ESM Fig. 3). At week 4, the hunger rating was statistically significantly reduced in the YesMilk group (*p*=0.025, within-diet, week 4 vs baseline) and was markedly lower than in the NoMilk group (*p*=0.001, between diets at week 4), driven mainly by lower post-lunch hunger (15:00–16:00 hours, *p*<0.05 within-YesMilk diet, week 4 vs baseline) (Fig. 5a, b and ESM Fig. 3a, b).

The fullness rating increased statistically significantly only in the YesMilk group (*p*=0.003, within-diet, week 4 vs baseline) (Fig. 5c). Although fullness appeared to be rated higher in the YesMilk group at the end of the intervention compared with the NoMilk group (ESM Fig. 3c, d), the between-group difference did not reach statistical significance (*p*=0.08, between diets, at week 4) (Fig. 5c, d). The desire to eat gradually declined throughout the intervention in the YesMilk group (*p*=0.0002, within-diet, week 4 vs baseline), resulting in lower scores compared with the NoMilk group at both time points (*p*=0.0036, *p*<0.001, between-diet, at week 2 and week 4, respectively) (Fig. 5e, f and ESM Fig. 3e, f).

Prospective consumption (participants’ estimated capacity to eat at a given moment) showed a similar pattern, as scores declined after 2 weeks of YesMilk diet (*p*=0.05, within-diet, week 2 vs baseline) and further by week 4 (*p*<0.001, within-diet, week 4 vs baseline), resulting in overall lower daily values compared with the NoMilk group (*p*<0.001, between diets at week 4) (Fig. 5g, h and ESM Fig. 3g, h). The desire for sweets decreased in the YesMilk group at both time points compared with baseline (*p*=0.0007, within-diet at week 2; *p*<0.001, within-diet at week 4), while no comparable changes were observed in the NoMilk group (Fig. 5i and ESM Fig. 3i, j). Overall, hunger and sweet craving scores decreased by 15–20% (*p*<0.05).

## Discussion

This randomised crossover study demonstrates that a dairy-based diet improves glycaemic management and circadian clock gene expression in individuals with type 2 diabetes. Glycaemic improvements were observed within 2 weeks and became more pronounced by 4 weeks, with fasting glucose decreasing by ~1.7 mmol/l, GMI from 6.8% to 6.1%, and mean daily glucose by >1.7 mmol/l. Notably, the most robust glycaemic reductions occurred during the afternoon and night-time, suggesting a prolonged metabolic effect initiated by morning dairy intake.

These results were consistent in both male and female participants and can be generalised to all genders in a population. This is the first clinical study to investigate how a dairy- vs non-dairy protein diet, structured around a chrononutritional pattern, affects circadian gene expression in white blood cells of individuals with type 2 diabetes. A key finding was the upregulation of *BMAL1*, *REV-ERBα*, *CRY1* and *PER1* in response to the dairy-based intervention, coinciding with reductions in fasting glucose, GMI and postprandial glucose, and increased TIR, suggesting a mechanistic link between protein source, nutrient timing and circadian regulation. Exploratory LMM results were directionally consistent with the paired *t* tests, indicating that treatment effects were not driven by dropout or sequence imbalance. Minor period effects observed for some glycaemic indices (TIR and TAR) likely reflected unequal sequence size rather than true carry-over. Overall, the absence of consistent period effects supports the robustness of our conclusions.

While whey protein acutely stimulates GLP-1 and insulin secretion [14, 21, 30], the marked nocturnal glucose reductions in the YesMilk group suggest effects beyond immediate hormonal responses. This aligns with the ‘second meal effect’, where a protein-rich breakfast, particularly whey, improves glucose levels at subsequent meals and during the nocturnal fasting period [5, 11]. As ~73% of daily dairy protein was consumed at breakfast, primarily as whey-rich liquid dairy, these effects likely reflect early-day metabolic priming and enhanced circadian alignment, highlighting the impact of breakfast composition on 24 h glucose homeostasis.

The YesMilk diet produced a distinct temporal response wherein *BMAL1*, *REV-ERBα* and *CRY1* increased after 2 weeks while *PER1* showed a delayed rise, significant only at 4 weeks. This staggered pattern suggests that dairy-derived nutritional cues may induce phase shifts in the circadian system, requiring sustained intake to fully engage and stabilise the molecular clock. These findings extend prior research on peripheral clock entrainment via meal timing and macronutrient composition [10, 31], highlighting dietary protein source, specifically dairy, as a modifiable factor influencing circadian alignment. Effects may be mediated by the amino acid profile of dairy proteins and bioactive peptides, which affect hormonal rhythms, serotonin production, insulin signalling and central clock pathways [15, 32]. Elevated *REV-ERBα* and *CRY1* expression has been linked to improved glucose homeostasis and suppressed hepatic gluconeogenesis, potentially contributing to the superior glycaemic outcomes seen with the YesMilk diet [33]. The structured meal schedule reinforces a stable feeding–fasting rhythm, known to entrain peripheral clocks, optimise hormonal coordination and enhance metabolic efficiency [34]. Thus, dairy protein may act not only as a metabolic substrate but also as a chrononutritional signal, strengthening the temporal structure of energy and nutrient intake in alignment with circadian biology.

These findings support evidence that circadian clock proteins directly regulate glucose metabolism. BMAL1 promotes insulin sensitivity and glucose homeostasis; its deficiency impairs glucose tolerance, and increases hepatic gluconeogenesis and pancreatic beta cell dysfunction [35]. REV-ERBα represses BMAL1 and modulates gluconeogenic enzymes and inflammatory pathways in the liver; its activation reduces hyperglycaemia in diabetic mice [36]. CRY1 regulates hepatic glucose production and insulin signalling; its overexpression has been associated with enhanced insulin sensitivity [37]. Thus, the upregulation of *CRY1* may underlie the reduction in nocturnal glucose levels observed in the YesMilk group. The delayed *PER1* upregulation may reflect longer-term adaptations in peripheral clock function. PER1 has been implicated in modulating both insulin action and glucocorticoid rhythms, with its disruption linked to metabolic inflexibility and impaired glucose clearance [38]. The coordinated upregulation of these genes suggests that the YesMilk diet may not only entrain the molecular clock but also enhance glucose regulation through multiple transcriptional pathways involved in hepatic glucose output, pancreatic function and systemic insulin sensitivity.

A key feature of the intervention was its meal timing structure, with a high-energy breakfast, substantial lunch and low-energy, carbohydrate-free dinner, aligned with circadian insulin sensitivity, which peaks in the morning and declines throughout the day [39]. Restricting carbohydrate intake to the early part of the day offers a practical adaptation of time-restricted eating (TRE) for real-world settings. Participants could eat until conventional dinnertime (~20:00 hours), creating a 12–14 h eating window but carbohydrates were limited to before lunch (~15:00 hours). This early carbohydrate-focused TRE mimics the metabolic benefits of early-TRE despite a longer eating window, potentially contributing to blunted evening postprandial glycaemic excursions. The YesMilk diet improved post-lunch and nocturnal glycaemic management, periods typically impaired in type 2 diabetes, suggesting that aligning dietary intake with endogenous circadian rhythms, supported by dairy protein, may enhance metabolic flexibility beyond macronutrient composition alone.

The YesMilk diet also improved appetite regulation. Participants reported lower hunger, reduced desire to eat or consume sweets, and greater satiety, especially in the afternoon and evening. These effects likely reflect both gut–brain hormonal responses and central appetite signalling. Dairy proteins stimulate GLP-1, peptide YY (PYY) and cholecystokinin (CCK), key satiety hormones, while tryptophan may enhance serotonergic activity, reducing reward-driven eating [30, 40, 41]. The slow digestion of casein may further prolong satiety and lower energy intake.

Our study has several limitations. First, the modest sample size (*n*=19 completing both arms) and relatively short intervention (4 weeks per condition) may limit generalisability and statistical power. However, carry-over effects were minimised by a 3–4 week washout period and further evaluated by comparing baseline CGM, fasting glucose, body weight and biochemical variables across phases and prior to the second intervention. Second, participants had well-controlled type 2 diabetes on stable oral medications, potentially reducing applicability to those with more advanced disease or insulin therapy. Third, gene expression was measured only in the fasting state and for a limited set of core clock genes in white blood cells, which serve as a proxy for peripheral clock activity but may not fully capture tissue-specific regulation. A transcriptome-wide approach in larger studies would provide valuable complementary insights. This study was designed as a mechanistic proof-of-concept study rather than a clinical implementation trial, aiming to test whether dairy-based protein modulates circadian clock gene expression and metabolic pathways in type 2 diabetes. While the findings may inform chrononutritional strategies, validation in larger, long-term clinical trials is needed before translation into dietary guidelines is possible.

In conclusion, a chrononutritional approach emphasising dairy protein, a high-protein breakfast and early daytime-restricted carbohydrate intake may improve glycaemic management, circadian alignment and appetite regulation in type 2 diabetes. Considering protein source, macronutrient distribution and meal timing offers a practical strategy to synchronise metabolic rhythms with nutritional cues. Future research should explore long-term adherence, diverse populations and interactions between chronotype, nutrient timing and disease progression. Personalised strategies that integrate both what and when we eat may be key to optimising glycaemic management in diabetes.

## Supplementary Information

Below is the link to the electronic supplementary material.

ESM (PDF 302 KB)

## Data Availability

The corresponding author will make data available upon reasonable request.

## Abbreviations

BMAL1: Brain and muscle ARNT-like protein 1
CGM: Continuous glucose monitoring
CRY: Cryptochrome circadian regulator
GLP-1: Glucagon-like peptide-1
GMI: Glucose management indicator
LMM: Linear mixed-model
PER: Periodic circadian regulator
REV-ERB: Nuclear receptor subfamily 1 group D
TAR: Time above range
TIR: Time in range
TRE: Time-restricted eating
VAS: Visual analogue scale
