# Supplementary material for: Glycaemic, appetite and circadian benefits of a dairy-enriched diet with high-protein breakfast and early daytime-restricted carbohydrate intake in type 2 diabetes: a randomised crossover trial
Source: Diabetologia. 2026 Jan 23;69(4):1021–34. doi: 10.1007/s00125-025-06658-2 (PMC12957644; doi:10.1007/s00125-025-06658-2)
Supplement: Supplementary file 1 — ESM (PDF 302 KB) [file 125_2025_6658_MOESM1_ESM.pdf]

ESM Figure 1

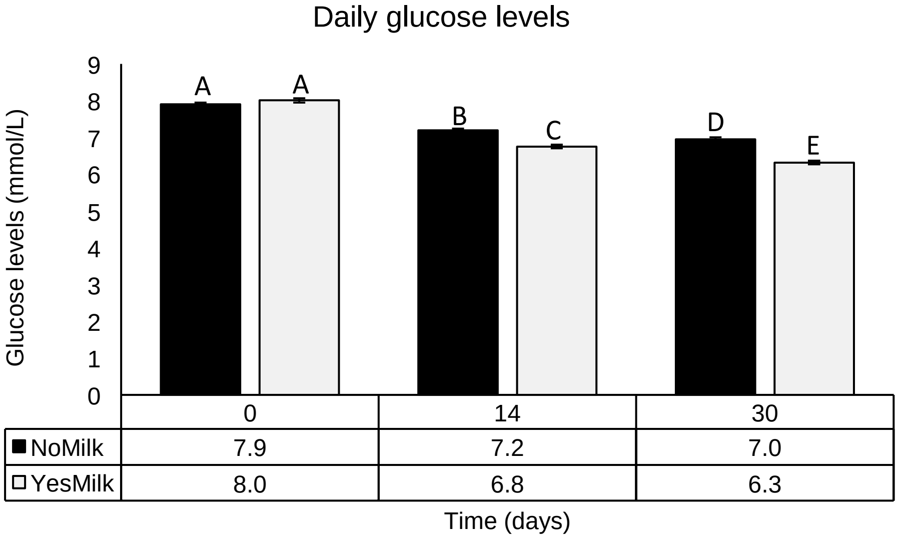

ESM Figure 1. Verification of average daily glucose levels using a custom CGM algorithm. Daily glucose concentrations (mmol/L) were calculated using hourly CGM data for each participant across the dietary intervention. These values were independently derived using a semi-automated algorithm developed for the study, which averaged glucose values for each patient for a 24-h period. Results were consistent with the LibreView software derived 24-h averages (see Fig. 2D), confirming the robustness and reproducibility of CGM-derived outcomes. Different letters indicate significant differences at $p < 0.05$ based on Tukey's HSD.

ESM Figure 2

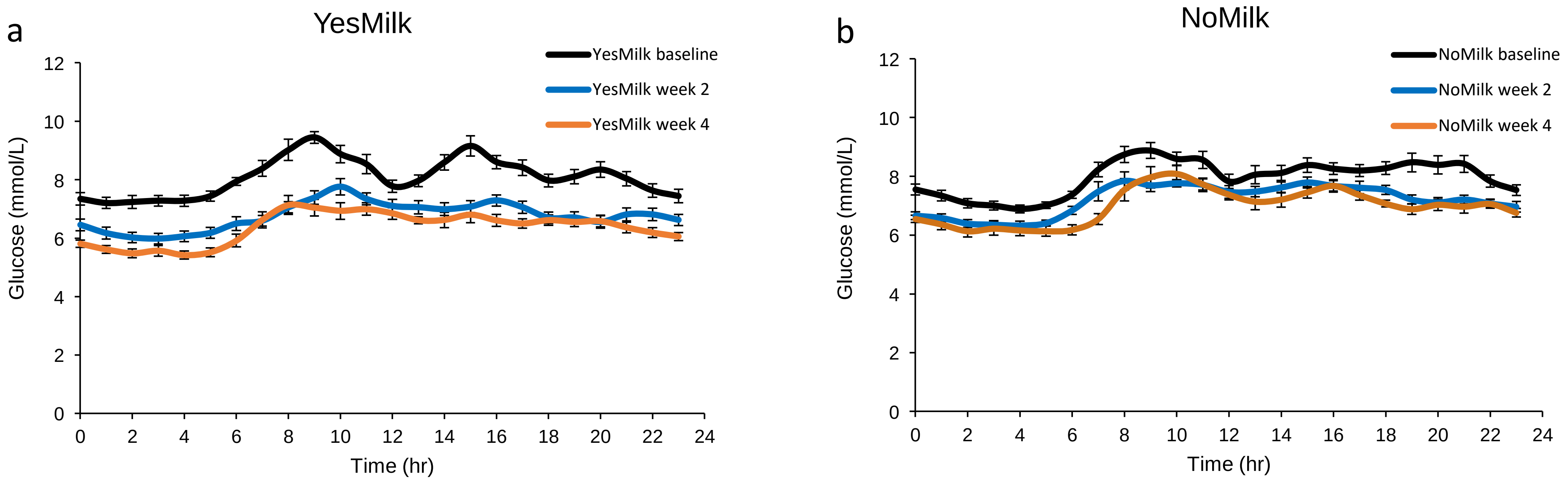

ESM Figure 2. Diurnal glucose profiles at weeks 2 and 4 of intervention.
(a) YesMilk group: glucose levels further decreased from week 2 to week 4, particularly in the afternoon (16:00–17:00) and during the night (22:00–05:00).
(b) NoMilk group: no significant changes in diurnal glucose profiles between weeks 2 and 4.

ESM Figure 3

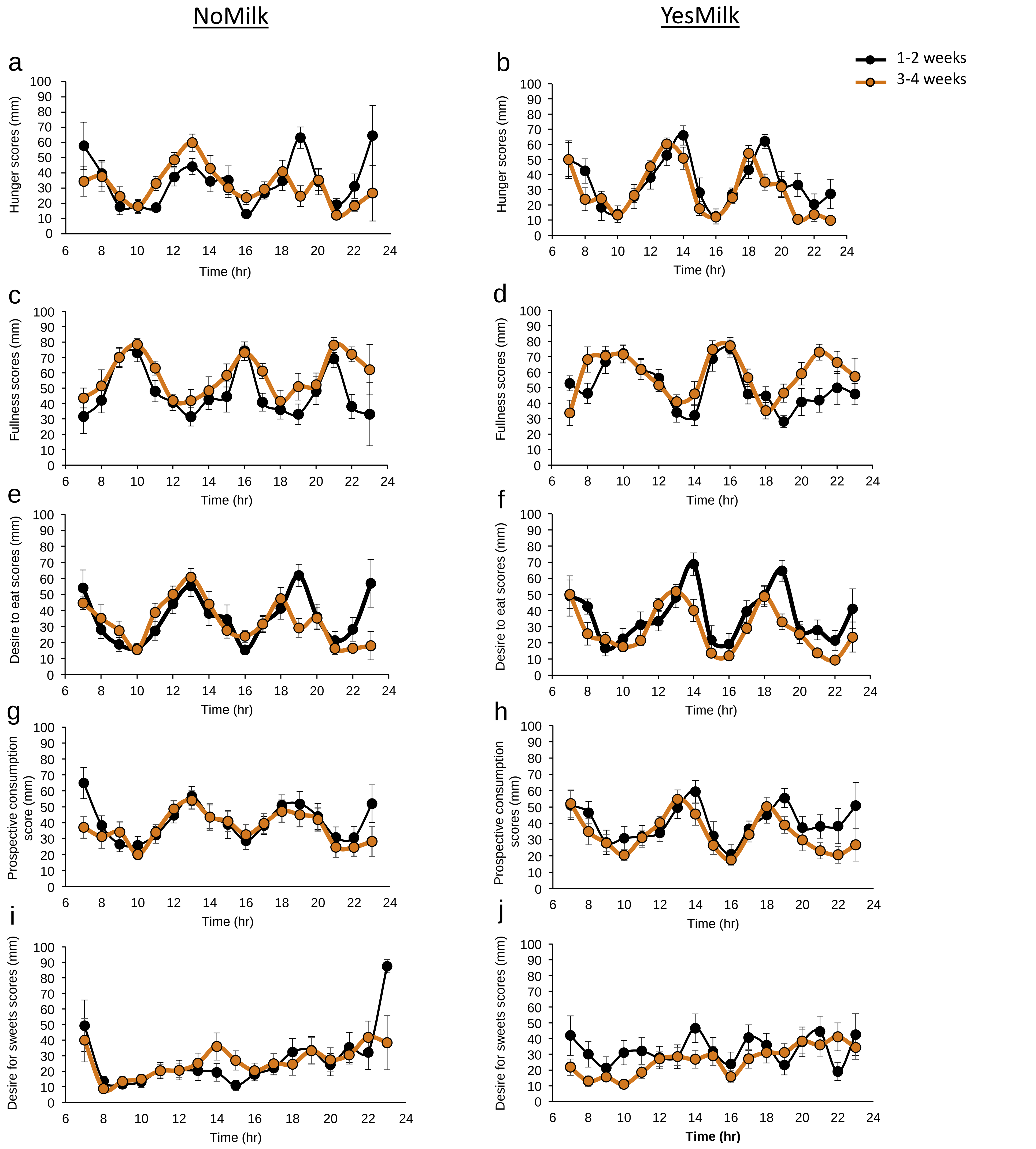

ESM Figure 3. Diurnal patterns of subjective appetite ratings across intervention weeks. Visual Analog Scale (VAS) scores recorded hourly over a 24-hour period in the NoMilk group (a, c, e, g, i) and the YesMilk group (b, d, f, h, j) after 1–2 weeks (black lines) and 3–4 weeks (red lines) of dietary intervention. Panels represent: (a-b) Hunger, (c-d) Fullness, (e-f) Desire to eat, (g-h) Prospective consumption, (i-j) Desire for sweets. Scores reflect mean ± SEM. Reductions in hunger, desire to eat, prospective consumption, and desire for sweets were more pronounced in the YesMilk group over time, particularly during the afternoon and evening periods.

ESM Table 1: Diet composition

	Goal		Carbohydrates	Fat	Protein
	**kJ (kcal)**	**%**	**% of energy**	**% of energy**	**% of energy**
**Breakfast**	3765 (900)	45%	50%	45%	45%
**Lunch**	3347 (800)	40%	40%	35%	35%
**Dinner**	1255 (300)	15%	10%	20%	20%
**Total**	8368 (2000)	100%	100%	100%	100%

ESM Table 2: Quantitative real-time PCR primer sequences

Gene	Forward primer	Reverse primer
ACTIN	5’-CAGGCACCAGGGCGTG-3’	5’-CCCACATAGGAATCCTTCTGACCA-3’
BMAL1	5’-ATTGAAACACCTCATTCTCAGGG-3’	5’-CCTACGACAAACAAAAATCCATCT-3’
CLOCK	5’-ACAGCTGCTGAGAAAAGCCAA-3’	5’-TGTGTTTATACGATTATCTGACCCAGAA-3’
CRY1	5’-GAAGTTGCTCTCAAGGGAGTGG-3’	5’-GACTGTCGCCATGAGCATAGTG-3’
RORα	5’-CAGGAAGAAATTGAGAACTATC-3’	5’-GCCACATCACCTCCCGCTGCTTG-3’
REV-ERBα	5’-CCAAGTCACCCTGCTTAAGGC-3’	5’-AAGCGCACCATCAGCACC-3’
PER1	5’-GGACCGACCCCTCATGCT-3’	5’-CCCGCCAACTGCAGAATCT-3’

ESM Table 3: Linear mixed model (LMM) analysis of treatment, time, period, and treatment×time effects for metabolic and clock gene outcomes.

Outcome	Fixed effect	F (df1,df2)	F ratio	P value	Random effect variance (%)	Residual (%)	Wald p value
**GMI**	Treatment	F(1,88.1)	10.06	0.0021	40.4	59.6	0.0165
	Time	F(2,88.1)	100.25	<0.0001			
	Period	F(1,88.1)	0.25	0.6165			
	Treatment * Time	F(2,88.1)	7.71	0.0008			
**Fasting glucose**	Treatment	F(1,87.4)	2.59	0.1111	36.1	63.9	0.212
	Time	F(2,87.3)	51.68	<0.0001			
	Period	F(1,87.4)	0.073	0.7876			
	Treatment * Time	F(2,87.3)	4.027	0.0212			
**Average glucose**	Treatment	F(1,86.7)	9.71	0.0025	39	61	0.0198
	Time	F(2,86.8)	100.011	<0.0001			
	Period	F(1,86.7)	0.082	0.775			
	Treatment * Time	F(2,86.8)	8.215	0.0005			
**Time in range**	Treatment	F(1,88.1)	0.031	0.8591	28.7	71.3	0.0356
	Time	F(2,88.2)	25.98	<0.0001			
	Period	F(1,88.1)	6.899	0.0102			
	Treatment * Time	F(2,88.2)	3.0879	0.05			
**Time above range**	Treatment	F(1,88.1)	0.239	0.6255	26.9	73.1	0.0411
	Time	F(2,88.2)	37.215	<0.0001			
	Period	F(1,88.1)	4.878	0.0298			
	Treatment * Time	F(2,88.2)	2.715	0.0718			
BMAL1	Treatment	F(1,76.3)	1.028	0.3137	21.3	78.7	0.1093
	Time	F(2,75.8)	3.976	0.0228			
	Period	F(1,76.3)	0.023	0.8797			
	Treatment * Time	F(2,75.8)	4.316	0.0168			
REVERBα	Treatment	F(1,77.8)	13.077	0.0005	30.7	69.3	0.0364
	Time	F(2,77.7)	2.328	0.1042			
	Period	F(1,77.9)	2.85	0.0953			
	Treatment * Time	F(2,78)	5.052	0.0086			
CRY1	Treatment	F(1,84.5)	1.814	0.1816	47.5	52.5	0.012
	Time	F(2,84.4)	0.108	0.8974			
	Period	F(1,84.4)	0.421	0.5182			
	Treatment * Time	F(2,84.2)	1.146	0.3227			
PER1	Treatment	F(1,83)	4.516	0.0365	45.7	54.3	0.0143
	Time	F(2,83.1)	0.8299	0.4396			
	Period	F(1,83.1)	0.0085	0.9264			
	Treatment * Time	F(2,83.1)	2.739	0.0704			

ESM Table 4: Daily Dietary intake of YesMilk and NoMilk diet interventions

Parameter	YesMilk	NoMilk	*p* value
**Energy [kJ (kcal)]**	8464 ± 209 (2023 ± 50)	8456 ± 167 (2021 ± 40)	0.9738
**Protein (g)**	139 ± 21.25	139 ± 19.81	0.9636
**Carbohydrates (g)**	152 ± 5	158 ± 5	0.4185
**Fat (g)**	89.8 ± 4.7	87.9 ± 3.2	0.7519
**Fibre (g)**	28.6 ± 1.9	29.8 ± 1.6	0.6435
**Calcium (mg)**	1200 ± 51	1211 ± 69	0.9044

ESM Table 5: Dietary intake at baseline

	YesMilk	NoMilk	*p* value
**Energy [kJ (kcal)]**	9791 ± 439 (2340 ± 105)	10079 ± 632 (2409 ± 151)	0.7085
**Protein (g)**	111 ± 7	114 ± 8.6	0.7813
**Carbohydrates (g)**	218 ± 15	233 ± 21	0.5626
**Fat (g)**	110 ± 8	105 ± 9	0.6882
**Fibre (g)**	32 ± 4.3	28 ± 3	0.4907
**Calcium (mg)**	815 ± 76	853 ± 154	0.8282

ESM Table 6: Reported dietary intake by group and meal type

Meal type	Parameter	YesMilk	NoMilk	*p* value
**Breakfast**	Weight (g)	879 ± 28	859 ± 29	0.6151
	Energy [kJ (kcal)]	3661 ± 117 (875 ± 28)	3745 ± 100 (895 ± 24)	0.5897
	Protein (g)	58.7 ± 2.8	59.2 ± 13.9	0.8941
	Carbohydrates (g)	69 ± 3.5	72 ± 3.7	0.4903
	Fat (g)	40.6 ± 2	40.3 ± 2.2	0.9063
	Fibre (g)	13 ± 1.6	15.3 ± 1	0.2644
	Calcium (mg)	722 ± 45	869 ± 48	0.0315
**Lunch**	Weight (g)	794 ± 47	747 ± 45	0.4736
	Energy [kJ (kcal)]	3264 ± 105 (780 ± 25)	3347 ± 134 (800 ± 32)	0.6184
	Protein (g)	50 ± 1.6	51.7 ± 2.6	0.5345
	Carbohydrates (g)	70 ± 4.1	73.5 ± 4.2	0.5724
	Fat (g)	29.2 ± 2	30.4 ± 2	0.6945
	Fibre (g)	11.2 ± 1	10.5 ± 1.1	0.6164
	Calcium (mg)	214 ± 18	227 ± 25	0.6889
**Dinner**	Weight (g)	469 ± 24	439 ± 18	0.32
	Energy [kJ (kcal)]	1577 ± 100 (377 ± 24)	1469 ± 84 (351 ± 20)	0.41
	Protein (g)	31 ± 2.2	31 ± 1.9	0.965
	Carbohydrates (g)	14.6 ± 1	12.2 ± 1	0.078
	Fat (g)	20.6 ± 1.8	19 ± 1.8	0.5184
	Fibre (g)	4.4 ± 0.5	4.3 ± 0.3	0.842
	Calcium (mg)	265 ± 29.3	135 ± 17.6	0.0007

ESM Table 7: Servings per day of different food groups

	Dairy products (unsweetened)	Dairy products (unsweetened low fat)	Dairy products Sweetened with Sugar	Fluid dairy	Non-fluid dairy products	Total dairy	Total non-dairy protein	Total non-dairy protein (including legumes)	Vegetables	Fruits	Total fats	Total grains
**YesMilk Baseline**	3.14	0.08	0.00	1.02	2.15	3.16	3.83	3.99	3.56	1.69	10.27	10.27
**NoMilk Baseline**	3.60	0.00	0.00	0.99	2.61	3.60	3.92	3.98	3.89	3.16	9.85	9.93
**YesMilk Diet**	4.76	0.42	0.23	1.61	3.66	5.27	5.68	6.10	7.90	1.42	6.47	4.83
**NoMilk Diet**	0.00	0.00	0.00	0.00	0.00	0.00	10.27	10.38	7.45	1.41	7.19	5.38

ESM Table 8: Protein consumption per meal (% of daily servings) during the YesMilk diet intervention

Meal/Protein classification	Fluid dairy	Non-fluid dairy	Total dairy	Eggs	Chicken and Turkey	Meat and Fish	Legumes	Plant-based dairy substitutes	Total non-dairy proteins	Total non-dairy proteins (including legumes)
**Breakfast (%)**	85	68	73	69	3	42	6	86	41	39
**Lunch (%)**	11	1	4	2	72	32	61	0	32	34
**Dinner (%)**	4	31	23	28	25	27	33	14	26	27
